# Pulp sensibility tests responses in patients with anxiety and depression

**DOI:** 10.4317/jced.59242

**Published:** 2022-05-01

**Authors:** Narges Farhad-Mollashahi, Mahboubeh-Firouzkouhi Moghadam, Seyed-Mohammad-Javad Aslani, Forugh Mollashahi

**Affiliations:** 1 Associate Professor, Oral and Dental Disease Research Center, Department of Endodontics, school of Dentistry, Zahedan University of Medical Sciences, Zahedan, Iran; 2 Department of Psychiatry, Research Center for Children and Adolescents Health (RCCAH), Zahedan University of Medical Sciences, Zahedan, Iran; 3 Resident of Endodontics, School of Dentistry, Zahedan University of Medical Sciences and Health Services, Zahedan, Iran; 4 Dentist, Zahedan, Iran

## Abstract

**Background:**

In view of the importance of pulp sensibility tests in clinical decision-making and the impact of psychological factors on test results, we evaluated in this study dental pulp responses to pulp sensibility tests (ie, cold and electric) in patients with anxiety and depression.

**Material and Methods:**

A number of 90 people age 20 to 30 participated in the study, including 30 healthy and 60 with anxiety and depression, whose disorder was approved by a psychiatrist based on the Symptom Checklist 90-R questionnaire. Pulp sensibility tests included electric and cold ones were performed on lateral mandibular teeth. The cold test results were recorded based on the visual analogue scale (VAS) pain scoring (0 no response, 10 worst pain). Electric pulp test was performed using a digital pulp tester. The lowest current that stimulated a pulp response was recorded. The data were analyzed using the Mann-Whitney and Kruskal-Wallis tests.

**Results:**

The cold test pain intensity was significantly higher in patients than in healthy subjects and was significantly associated with the severity of anxiety and depression. In addition, the electric pulp test current to evoke a response was significantly lower in patients than in healthy subjects and was also significantly associated with the severity of anxiety and depression.

**Conclusions:**

Given the limitations of this study, anxiety and depression significantly affect the results of pulp sensibility tests.

** Key words:**Anxiety, Cold pulp test, depression, electric pulp testing.

## Introduction

Pulp sensibility is an important part of diagnosis when assessing pulp health. Thermal and electrical tests are the most common pulp sensibility tests in assessing the condition of the sensory nerve fibers (nociceptors) of the pulp (1). These tests have been shown to be accurate and reliable in distinguishing between vital and non-vital pulps. Numerous factors influence the diagnostic accuracy of the tests (2,3). The response of the dental pulp to the nociceptor stimulus varies from person to person and is influenced by various factors such as gender. Women are more susceptible to pain than men. Studies suggest that gender plays a role in pulp sensitivity, although not unanimously (4).

Psychological, genetic, and chronic pain factors also play a role in a person’s response to pain. The perception of stimuli is related to numerous psychological factors such as psychosocial stress, emotional distress, anxiety, depression and somatization (5). Both depression and somatization can play a role in clinical pain intensity in people with chronic pain (6). However, their association with pulp sensibility has not been established. Klauenberg *et al*. showed that depressive symptoms may intensify pain (7). In a study, people with higher degrees of depression had lower electric pulp test response and higher cold test pain intensities (8).

Given the importance of pulp sensibility tests in clinical decision-making and the impact of psychological factors on test results, as well as the lack of studies on the subject, in this study we evaluated the response to pulp sensibility tests in patients with depression and anxiety.

## Material and Methods

A number of 90 people age 20 to 30 participated in the study, including 30 healthy and 60 with anxiety and depression, whose disorder was approved by a psychiatrist based on the Symptom Checklist 90-R questionnaire. The study protocol was approved by the ethics committee (no:IR.ZAUMF.REC.1400.030) and written consent was obtained from the participants. Inclusion criteria were lateral mandibular teeth free of caries, restoration, crowns, veneers and abrasions, with no signs of pulp and periapical disease based on clinical and radiological evaluations, and no history of trauma and recent orthodontic or periodontal treatment. Exclusion criteria were systemic disease, use of pacemaker, pregnancy and breastfeeding, use of contraceptives, irregular menstrual cycles, calcified teeth and use of drugs that affect pain perception in the last 24 hours and 

Pulp sensibility tests, including electric and cold ones, were performed on patients after confirmation of their disorder by a psychiatrist and before taking sedatives and anti-anxiety medications. The tests were carried out on lateral mandibular teeth. The tooth surface was isolated with celluloid matrix tape and dried with a cotton swab prior to test. A digital pulp tester (Parkell, Brentwood, NY) was used for the electric pulp test. A thin layer of toothpaste (Pooneh, paksan, Tehran, Iran) was placed The probe was placed on 1/3 of the incisal of the buccal surface in the cervical of the tooth, and the lip clip of the pulp tester was placed in the individual’s mouth. Thereafter, the patient was asked to inform the examiner whenever he or she felt tingling, pain, or any sensation during activation of the electric pulp test. The lowest current that stimulated the pulp response was considered the patients’ response.

A cold spray (Luber Cool; Marcadent, Tehran, Iran) was used for the cold test. After isolating the teeth with a cotton roll and drying the tooth surface, a size 2 cotton pellet was sprayed and placed at the center of the buccal surface for 5 seconds or as soon as the patient raised his or her hand to indicate feeling either pain or a cold sensation. The severity of pain was rated based on VAS in the range 0-10 (0 no response, 10 worst pain). In the case of no response, the test was repeated. Recovery period was about 2 minutes between sensibility tests. The data were analyzed by SPSS 20.0 using the Mann-Whitney U and Kruskal-Wallis tests.

## Results

There was no significant difference between the age and sex of the participant in two groups (Table 1).

**Table 1:**
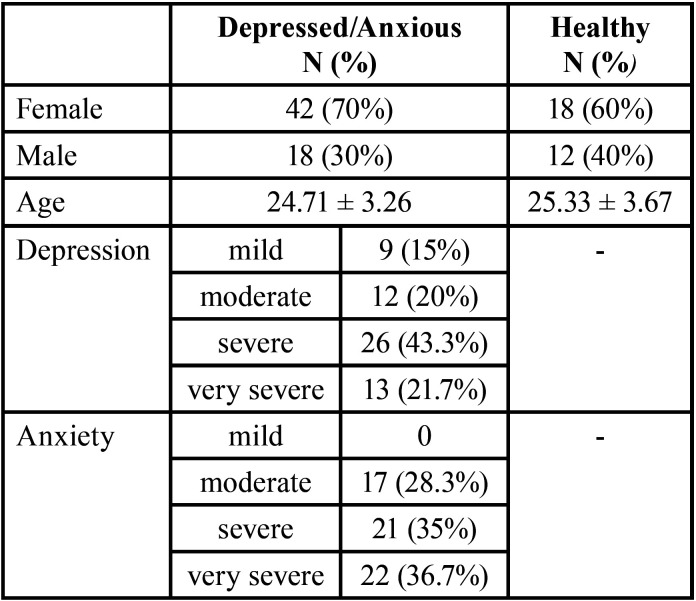
Demographic information of the participants.

According to Table 2, the cold test pain intensity was significantly higher in Depressed/Anxious patients than in healthy individuals, whereas the electric pulp test current to evoke a response was significantly lower.

**Table 2:**
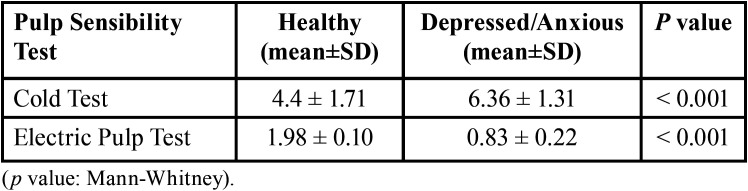
A comparison of the responses to pulp sensibility tests in patients and healthy group.

As given in Table 3, the cold test pain intensity was significantly associated with the severity of anxiety and depression, as patients with severe or very severe disorders experienced more pain compared to those who had mild or moderate disorders. Moreover, in people with severe or very severe anxiety or depression, the electric pulp test response was significantly lower than in people whose disorder was mild or moderate.

**Table 3:**
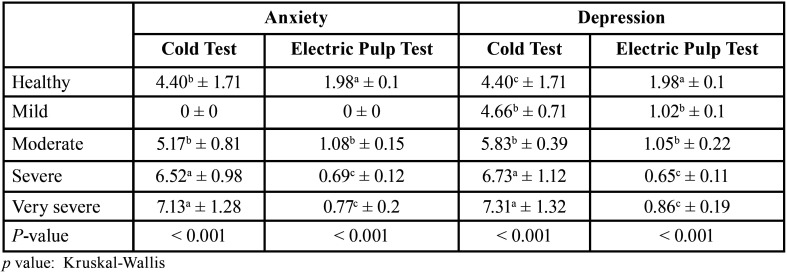
Responses to cold and electric pulp tests according to the severity of disorder. Data are represented as mean ± SD.

## Discussion

Dental pulp testing is a useful and essential diagnostic aid in endodontics. Pulp sensibility tests include thermal and electric tests, which extrapolate pulp health from sensory response. Whilst pulp sensibility tests are the most commonly used in clinical practice, they are not without limitations and shortcomings. Since pulp is surrounded by hard tissue, direct observation prior to endodontic treatment is not possible. Therefore, indirect methods are used to examine the pulp. The most common tests are thermal and electrical ones (44). The results of a meta-analysis showed that the cold test is the simplest and most accurate pulp sensibility test available to clinicians as a primary diagnostic tool. The electric pulp test has low sensitivity and high specificity and is suitable for the detection of vital pulp (45). Numerous factors, including psychological ones, influence the diagnostic accuracy of pulp sensibility tests (2,3). Both depression and somatization can play a role in the clinical assessment of pain in people with chronic pain (6), although their association with pulp-sensitive reactions has not been established.

The aim of this study was to investigate the effect of anxiety and depression on pulp sensibility tests. A distinction between anxiety and depression in patients is not possible, as these two disorders mostly occur together, according to studies and reference texts. Studies show that 91% of patients with anxiety have at least one other psychotic disorder. About one-third of people with anxiety develop severe depression before the disorder begins, and another two-thirds experience anxiety during or after depression (48,49). Therefore, the patients in this study were assigned to only one group and categorized according to the severity of their disorder. We examined 60 people with anxiety and depression and 30 healthy subjects. The results showed that there was a significant difference in responding to cold and electric pulp tests between patients and healthy individuals and was also significantly associated with the severity of anxiety and depression.

The response of the dental pulp to nociceptor stimulus varies from person to person and is influenced by gender. Women are more susceptible to pain than men. Although not supported by others, some studies suggest that gender plays a role in pulp response (4).

According to a 2018 study by Mladenovic *et al*. when examining the effect of psychosocial factors on pulp sensibility, people with higher degrees of depression showed lower electric pulp test response and higher cold test pain intensities (8). Their results agree with those of the present study. In addition, they found that the pulp sensibility tests was influenced by gender. In our study, anxiety and depression were also more common in women.

Shang *et al*. showed that chronic pain is often associated with persistent anxiety (41). Different cold test responses have been reported in patients with chronic facial pain. In the Mladenovic’s study, the pain intensity was higher in women with temporomandibular disorders (a chronic painful condition) on the cold test (8). Lower electric pulp test thresholds and higher cold test pain intensities were also observed in people with higher degrees of depression or somatization (8).

Edwards *et al*. showed that the pain perception threshold and pain tolerance were higher in healthy women compared to healthy men. However, in patients with pulpitis, men and women responded similarly to thermal tests in terms of pain intensity. Patients with pulpitis were also more anxious than the control group (42).

In the present study, the electric pulp test threshold in people with anxiety and depression was significantly lower than in healthy ones. In addition, the threshold decreased as the severity of anxiety or depression increased. Electric pulp testers stimulate myelinated A-delta nerves and have no effect on C nerves due to their higher stimulation thresholds. It has been shown that in pulpal disease due to caries or trauma, the electric pulp test does not indicate the severity of disease; rather it only indicates the pulp’s status, i.e., vital or necrotic. During the electric pulp test on a tooth with a normal pulp, the results are actually the response of the pulp nerves to electrical current. Some factors such as caries, extensive dental restoration, and periodontal disease can affect the pulp’s response to sensibility tests (50,51). To eliminate the effect of such factors, teeth with extensive restoration, tooth decay, and periodontal disease were therefore excluded in this study. To this end, a thorough clinical examination was performed and periapical x-rays of the target teeth were obtained.

According to Dworkin *et al*., a controlled psychological preparation (anxiety reduction) before administering usual nitric oxide doses can significantly alleviate pulpal pain (43). Farid *et al*. showed in a correlation study that mood and personality characteristics influence pain intensity and persistence. However, between anxiety and depression, the severity of pain and its interference with daily activities can only be predicted by anxiety (39).

Systemic diseases can also affect the response to sensibility tests. For example, a stronger electrical stimulus is required to stimulate the pulp in patients with hypertension or hyperparathyroidism compared to healthy individuals (54-52). To eliminate the impact of this factor on the results in this study, patient blood pressure was recorded and suspect patients were excluded. Also, given the effect of some drugs, in particular, Non-steroidal anti-inflammatory drugs(NSIAD) and analgesics on results (52,53), participants were asked not to take such drugs in the last 24 hours prior to study.

We observed no significant difference between the groups in terms of age. To obtain more reliable results in responding to pulp tests, subjects aged 20-30 years were selected. Indeed, pulp sensibility decreases in the elderly due to the decreases in pulpal nerves as a result of the reduction in the size of the pulp chamber (55). On the other hand, studies on the sensibility of the pulp during the growth and maturation of the tooth show a decrease in the stimulation threshold in this phase (56). It has been shown that the response of young incisor teeth to electric pulp test increases as the tooth root develops (57).

The results of this study showed that personal characteristics and mood traits influence pain intensity and pain threshold. Therefore, caution should be exercised in clinical judgments when evaluating pulp sensibility tests in patients with anxiety or depression.

## Conclusions

The pulp sensibility tests may be influenced by anxiety and depression. Lower electric current was needed to evoke a pulp response. The pulp response to the cold test was intensified.
